# Customer knowledge management competence evaluation of agritourism enterprises by using the balanced scorecard and fuzzy-AHP: Evidence from Chengdu-Chongqing economic circle

**DOI:** 10.1371/journal.pone.0280482

**Published:** 2023-02-09

**Authors:** Guoyi Chen, Jiansheng Zhang, Wei Tan, Shangmin Zhang, Bangquan Yan

**Affiliations:** 1 School of Business Management, Chongqing Three Gorges University, Wanzhou, Chongqing, China; 2 Scientific Research Department, Chongqing Three Gorges University, Wanzhou, Chongqing, China; 3 School of Public Management, Chongqing Three Gorges University, Wanzhou, Chongqing, China; Universita degli Studi del Molise, ITALY

## Abstract

This paper provides an evaluation framework to explore the linking mechanisms between customer knowledge management competence (CKMC) and Balanced Scorecard (BSC). With a case study from Chengdu-Chongqing Economic Circle of China, this paper attempts to empirically justify the framework. An index system was established for evaluating CKMC based on BSC and knowledge management process, the weight design and consistency check of the indexes were implemented by using the analytic hierarchy process (AHP), and the overall evaluation value and concrete index scores at all levels were obtained via the fuzzy evaluation method. Empirical results show that CKMC performance measurement indicators were ranked in order of importance as Business process performance dimensions (0.465), System support dimensions (0.289), Customer communication dimensions (0.152) and Market performance dimension (0.094). It also shows that the overall score of CKMC was 3.404, reflecting that the CKMC was in a state of general satisfaction. This research also identifies key factors hindering implementation of CKMC, including Attention from senior leaders (2.871), customer knowledge sharing efficiency (2.928), and information technology level (3.133). This research could contribute to CKM theory by extending customer knowledge management competence research with BSC initiatively. For practitioners, this study may provide useful suggestions to identify key factors promoting business CKMC, and finally promotes sustainable development of Agritourism.

## Introduction

In recent years, agritourism has developed rapidly in China. According to the survey of National Bureau of Statistics of China, the number of Chinese farms providing agritourism and recreation services rose from 26,000 in 2010 to 216,000 in 2019, an increase of 26.5% [1]. And it has played an increasingly important role for social-economic development in the rural areas, especially for those poverty-stricken area in western China. However, after 2020, The COVID-19 pandemic has dramatically impacted tourism and leisure activities worldwide, especially in the agritourism sector, the downsizing strategies of agritourism enterprises have caused an irrecoverable loss of intangible assets [2]. Under such circumstance, customer knowledge management (CKM), focusing on customer knowledge acquirement, transfer, sharing and creation, has received substantial attention from public.

In fact, since 2000, with the emergence of knowledge economy and widespread of information technology, business organizations were faced with the rapid change of market competition environment and the gradual deepening of global economic integration, which prompts the business to transform from product-centered to customer-centered business model, and focusing on customers’ demand and listening to their voice [3]. Organizations traditionally relying on internal knowledge have conducted a strategic shift toward customers, who are considered as valuable resource [4]. This led to the wide spread of Customer Knowledge Management (CKM) in both business and academic fields. As the core part of knowledge management, CKM will not only improve the customer service quality of tourism enterprises, and more importantly, it assists agritourism business to retain and create customers by integrating, sharing, and applying the customer knowledge, and to foster sustainability-oriented service innovation through customer knowledge innovating [5, 6], and finally improve their performance [7].

CKM must be effectively carried out based on the scientific and feasible evaluation of management performance [8]. Then how to reasonably develop, utilize and evaluate CKM become an important topic. However, literature suffers from a lack of understanding of customer knowledge management competence evaluation, especially in traditional agritourism industry. In the past, increasingly more qualitative researches have focused on the application and development of customer knowledge management (CKM) in the IT-based large-scale companies while neglecting its application in rural organizations, which is considered as huge blue sea market in China. Similarly, Balanced Scorecard (BSC) has also been widely applied for performance evaluation in industrial organizations, while few had been used in Chinese traditional rural organizations. Secondly, although, more and more western scholars have focused on CKM implementation and adopt new measurement techniques to measure CKMC, and most evaluation index were identified and discussed, but their explanatory power for the performance evaluation of CKMC is limited [4]. In details, past researches adopt eco-nomic value added (EVA) and key performance indicator (KPI), etc. to evaluate CKMC performance, but these evaluation systems are more or less one-sided, along with various problems such as incomprehensive index system, poor index representativeness and poor operability [9]. Thirdly, the researches on the performance evaluation of CKMC at the level of specific industries or enterprise application have not been unfolded, yet, and they mostly rest on theoretical discussion and model construction [4, 9]. The researches about application and performance evaluation of CKMC in Chinese agritourism is relatively few. In general, past researches of CKMC are focusing on one-sidedness, lacking depth and breadth, they were evaluated to measure IT industries by western scholars, the application and evaluation of CKM in agritourism is quietly few [10].

The goal of the research was to understand how customer knowledge is used in agritourism organization. Based on the theoretical framework of BSC, this paper combined KM with CRM to construct CKMC evaluation index system, and conducted CKMC evaluation by using Fuzzy-AHP comprehensive analysis, a case was adopted to validate the reliability and accuracy of this evaluation system.

This study provides new insight into customer knowledge management for agritourism managers and decision-makers under Digital Economy Era. Through CKMC evaluation, the level I and level II influence factors were identified and analyzed, and a performance evaluation framework was established. Through effective adoption of CKMC, it is expected that the findings will promote knowledge flow and customer maintain in the business. Besides this, this study is expected to contribute to academic literature on the traditional agritourism field in China. It has initiatively established an index system for customer knowledge management competence evaluation, it will enrich the theoretical foundation for CRM and KM theory, and secondly it has verified the index system through fuzzy AHP analysis based on a case study, thus it is beneficial for business knowledge management practices. Thus, the research of CKMC is of both academic and practical value for business professionals.

## Literature review

### Customer knowledge management

Customer knowledge management (CKM) mainly deals with customer knowledge, which was noted as the continuous dynamic combination of experience, perception, value and information created and absorbed in the interaction between customers and organizations [11]. Application of this knowledge is beneficial for conducting sustainability-oriented service innovation and enhance productivity and efficiency of modern tourism industry [5]. And this knowledge is categorized into three classic types including Knowledge from Customer, Knowledge for Customer and Knowledge about Customer. And Recently there is also the knowledge “with” customer [12].

The emergence of CKM is based on the concept of transforming customers’ role from passive marketing objects into actively knowledge contributors through interactive communication and collaborative innovation. Customers are those who know well of what they want and need, and they also best know how to express their needs to the business with organizations rational, emotional, and spiritual knowledge [13], whereas they do not know the business capacity of satisfying their needs, thus the knowledge from the customers component should be guided by managers, through knowledge and knowledge management.

CKM was initiatively considered as the external perspective of knowledge management, which assist’ to increase organizational absorptive capacity and enhance mutual relationship between customers and organizations [14]. In sequence, several researches conducted by [4, 15, 16] stated that CKM was the integration of CRM and KM, it indeed improves the understanding of customers’ need and demand through applying KM thought on CRM function extensions. Lak and Rezaeenour further added that both CRM and KM had actively played an important role in their respective field [13], this leads marketing professions to make best endeavors to integrate them together, as CRM is beneficial for customer interaction, and KM contribute to the acquiring, sharing and creation of customer knowledge to generated value.

### Customer knowledge management competence evaluation

Customer knowledge management (CKM) is a new management topic which emerges along with continuous development of the information technology, Knowledge Management (KM) and Customer Relationship Management (CRM). CKM is the strategic reflection of the numerous opportunities and challenge that the enterprises are facing [17]. It studies on how to gain, share, innovate and optimize the enterprise’s value chain by using customer knowledge effectively and meet customer demand for personalization customization, and then to display the leverage function of customer knowledge and the maximize efficiency of other resources of enterprise fully [18]. According to existing research, whether customer management is effective or not depends on Customer Knowledge Management Competence (CKMC). Therefore, CKMC studies have great theoretical significance and practical value.

In the past, numerous scholars have conducted researches on performance evaluation of CKMC, and achieved some important findings regarding the construction of evaluation index system and selection of evaluation method. Some main evaluation institutions and their evaluation contents and methods are listed in Table 1.

**Table 1:** Evaluation method and content of knowledge management.

Performance evaluation method	Evaluation institution or scholar	Main contents of performance evaluation
knowledge management assessment tool	American Productivity and Quality Center	Measure knowledge sharing and management quality from five dimensions: knowledge management process, leadership, organizational culture, technology, and performance evaluation [19]
MAKE (the most outstanding knowledge-based enterprise in the globe)	Teleos and knownetwork	Give scores using ten-grade scale based on Delphi method. The key dimensions include effort level made to create knowledge-based culture, recognition and support from senior leadership, ability to develop knowledge-based products, knowledge sharing measures, customer management capability, etc. [19]
Evaluation scale of knowledge management	David skyrme	Measure from 10 aspects: leadership, cultural environment, process, explicit knowledge and implicit knowledge, knowledge center, market effect, evaluation, personnel, technology, and scientific and technological base [19, 20]
Diagnostic tool of knowledge management	Bukowitz and Williams	Evaluate the knowledge management through information collection, information use, knowledge learning, evaluation, formal establishment, and exclusion of non-strategic knowledge [20].
Evaluation of knowledge value chain	ChingChy Lee	Evaluate the knowledge management from two major constituent parts of knowledge value chain: knowledge management base and knowledge management process [21]
Balanced score card (BSC)	Kaplan And Norton Tiwana	BSC method evaluates the knowledge capital from four aspects: finance, customer, enterprise internal operation, and learning and growth [22].
Data envelopment analysis (DEA)	Aim	The DEA method evaluates the performance of knowledge management [22]

It can be seen from Table 1, majorities of previous researches focus on knowledge management evaluation from two perspectives, including knowledge management process value chain and knowledge management base, while neglected the combination of knowledge management practices with strategy management. In fact, the effective adoption and implementation of KM requires a collective effort of the whole company, thus the comprehensive evaluation of knowledge management performance should take strategy management tool into consideration. In general, performance evaluation of CKMC has been systematically investigated in the academic circles, and numerous evaluation systems were identified. Due to the difference of different research samples, these researches are of very strong regional-ism, and the evaluation methods adopted by different scholars also proves to be quite different from each other to some ex-tent.

Although the researches on CKM have a late start in China, some findings and achievements have also been obtained. Generally Chinese scholars reviewed CKM mainly focus on the performance evaluation of CKM, including corporate-level knowledge management competence evaluation, knowledge management capability audit, and enterprise knowledge management system evaluation, etc. For instance, in [23], Zhao et al used the compound DEA method and combined the knowledge management practice to put forward methods for measuring and evaluating CKMC, and four dimensions were included for performance evaluation: leadership, organization, individual, and environment. In their research, the internal resources and capacities were all taken into consideration for comprehensively evaluating CKMC from the strategic perspective, it was of a certain reference value for studying overall CKMC, the main drawback lies in the lack of integration with specific knowledge management practice, thus the evaluation conducted by combining strategy management with specific customer knowledge process or customer ser-vice process, was of great importance. In [24], Wang M proposed a set of comprehensive evaluation index system for CKMC, which covers three dimensions: management process index, business outcome index, and organizational effect index, and a mathematical model for the comprehensive evaluation of enterprise CKM were constructed. This study introduced the quantitative evaluation index system of CKM, but neglected the combination with business practice, and was also proved weak in terms of empirical test. Subsequently, for addressing the deficiencies mentioned above, in [25], Li J et al. introduced the fuzzy multi-stage comprehensive evaluation, and constructed a comprehensive evaluation index system for enterprise knowledge management by selecting the following indexes: external structure, internal structure, and personnel competitiveness. When studying the relationship between knowledge management strategy and business performance, in [26], Chiu and Fogel put forward five-dimensions evaluation index for measuring CKMC compared with other important competitors, and the five dimensions included market share, growth rate, profitability, innovativeness, and enterprise scale change, majority of them are constructed based on the idea of BSC. Mathematical evaluation model were established in above researches, and evaluation was conducted based on expert experience and judgment, while considering the systematic and multi-faceted proper-ties of objects, this type of Expert Opinions could not make a comprehensive judgment of the performance results, resulting in a lack of objectivity for this method, which could be considered as the drawback of their researches.

Up to now, majority of the related studies focused on data model construction and simulation modelling [27], while few have been conducted in combination with practical operations, not to mention the empirical analysis. The evaluation systems are proved not perfect enough, the angles of thinking and indexes adopted vary from scholar to scholar, it is difficult to quantify multiple indexes, and there is no universal evaluation index system, which has been well-recognized and extensively promoted in the academic circles. The researches evaluating CKMC in tourism industries or have not been unfolded yet, and the accurate and mature performance evaluation index system for agritourism CKMC have not been established, either [28].

### Balanced scorecard and evaluation index

In an effort to develop a more complete measure of performance, in 1990, the Nolan Norton Institute, the research arm of KPMG, sponsored a multicompany project; "Measuring Performance in the Organization of the Future" [29] The results of the study were summarized in a 1992 article that introduced the BSC [29].

The organizational performance management method, balanced score card (BSC) proposed by Rober Kaplan from Harvard Business School and Director David Norton of Nolan Norton Institute [30], was used to measure the organizational performance. This BSC utilizes the vision and strategy from an organization’s stated mission to develop a comprehensive measure of its performance. The BSC incorporates a mixture of past performance measures with measures of the drivers of future performance. These measures enable the organization to be evaluated from four perspectives: (1) financial, (2) customer, (3) internal business processes, and (4) learning and growth. These four perspectives combine traditional short-term, financial-based measures, which indicate the organization’s performance over the past year, with non-financial measures, which are the assumed value drivers for the organization’s long-term financial performance [29, 31].

The research on the BSC has been vast since 2000. As noted previously, most studies have focused on either the implementation or the effects of implementing a BSC. Only a few projects have studied the BSC as a performance measurement tool. Some suggested areas of research that need to be addressed about the BSC as a performance measurement tool include the use of subjective versus objective measurements, the effects of using broad sets of metrics in performance measurement systems. and determining what ’`balance ’ is in the BSC [31, 32]. In fact, The Balanced Scorecard has been a major focus of performance evaluation literature in recent years. They deemed that the improvement in product quality, productivity, and customer satisfaction could bring benefits to the organization only after being transformed into the in-crease of sales volume, reduction of operating cost, and elevation of asset turnover ratio [30–32]. By reference to BSC, four indexes—business process, customer communication, system support, and market performance—were used in this research to measure the enterprise CKMC.

#### Business process index

The CKM activities include customer knowledge acquisition, sharing, application, and innovation [8], so the business process indexes should include: customer knowledge acquisition capability (CKAA), efficiency of customer knowledge sharing mechanism (ECKS), customer knowledge application capability (CKAC), and customer knowledge innovation capability (CKNC). The customer knowledge capability requires that the enterprise should have the basic quality management of acquiring the customer knowledge [33], and this quality is mainly manifested by the following aspects: enterprise operation and management level, technological level, capital strength, talent strength, etc. [34]. In the efficiency measurement of customer knowledge sharing mechanism, the customer knowledge coverage and reaction capacity to customer change should be mainly considered.

The customer knowledge application capability is expressed by two indexes: gold customer identification ability, 80% of an enterprise’s profit is created by 20% of its customers [35], and these customers are called gold customers to whom special attention and preferential should be paid and given; ability of understand customer purchase motive, the customer purchasing behaviors can be accurately predicted and guided only through the real understanding of customer needs and motives [36]. The customer knowledge in-novation capability is mainly reflected by two aspects: new product development capability and customized production capacity.

#### Customer communication index

The customer communication index is mainly manifested by four aspects: diversity (DIVE), timeliness (TIME), and effectiveness of business communication with customers (EBCC), and their ability to handle customer complaints (AHCC) [37]. The more the enterprise exchange modes with customers, the more convenient the communication will be; the timeliness of communication is embodied by two aspects: waiting time and customer abandonment rate during the exchange with the enterprise. The diversity and timeliness of communication provide a basis for maintaining the customers, and its effectiveness, which reflects the quality of customer relation, is generally reflected by the time spent by the enterprise to answer customer questions, and friendliness, alertness, and insight of communicating personnel. The ability to handle customer complaints refers to the speed and effectiveness of handling customer complaints, namely, the average time spent in solving the customer complaints, and the customer satisfaction with the solutions.

#### System support index

The system support index includes supporting capacity of organizational environment (SCOE), sustaining power of human capital (SPHC), information technology level (ITLE), and degree of importance attached by senior leadership (IASL) [38]. The CKM can be well supported from the organizational level only when the organization owns the strong adaptability to the environment. The sustaining power of human capital is reflect-ed by the proportion of knowledge employees, and employee training time and efficiency.

The advanced information technology will facilitate an enterprise to effectively man-age its customer knowledge, establish the knowledge management system, and improve its innovation capability. As an important reform of enterprise management means and method, the CKM can be smoothly carried out only with the support and assistance from the senior leadership. Therefore, the recognition level of senior managers for CKM and the popularization of CKM should be strengthened in order to put the CKM into practice.

#### Market performance index

The market performance index is reflected by four aspects: customer acquisition rate (CARA), customer satisfaction (CSAT), cross-sales volume (CSVO), and sales growth rate (SGRA). The customer acquisition rate refers to the proportion of target customers obtained by the enterprise. The customer satisfaction is associated with the value of products or services provided by the enterprise to customers. If the value is higher than the price of cost paid by customers, the customer satisfaction and loyalty can be improved [39]. The cross-sales volume reflects the quality of enterprise-customer relation, and it depends on the enterprise’s ability to communicate with customers, and ability to create and transfer the customer value, and these abilities will be key factors deciding the possibility of cross-purchase. The sales growth rate is the ratio of sales growth of the enterprise in this year to the gross sales in the previous year. Reflecting the change of enterprise sales revenue, the sales growth rate is an important index evaluating the enterprise growth status and development capability.

Through the adoption of the balanced scorecard at an agritourism company, the executives plan to give employees a clear and concise picture of the organization’s vision. The executives also plan to have the balanced scorecard be the basis of both the executive management team and board agendas. The President and CEO will help translate the vision into operational objectives so that every employee in the company will have detailed information about how they can carry out a plan that they fully understand.

Finally, Kaplan and Norton define the balanced scorecard as “a framework that helps organizations translate strategy into operational objectives that drive both behavior and performance” [40]. In this new management system, everyone understands where the organization is going and employees know in detail the vision and what specifically they can do to help the organization achieve the desired future. It is also believed that the balanced scorecard system will create an environment where the executives spend more time on strategy and less time on less important operational minutia.

### CKMC evaluation reviews gap

CKM has become the core of enterprise knowledge management activities, and its effective implementation must be based on the scientific and feasible management performance evaluation. Up to now, numerous researches have been conducted for evaluating CKMC in the academic circles, and increasingly more and more factors and dimensions were taken into consideration, However, because of different research settings and data processing methods, these researches are of very strong regionalism, most researches conducted in western countries cannot be easily applied in Chinese business organizations, especially in newest agritourism companies. The gaps identified in previous literature is twofold. On one hand, majority of Chinese researches were found to be dis-integration with customer knowledge management practice, the results and outcomes could not be combined with business CKM practice. And on the other hand, some researches adopted Delphi method as a data processing method to evaluate CKMC, which relied on experts’ opinions to measure CKMC, was proved to lack objectivity.

Given this, the evaluation index system for CKMC was built based on the knowledge management process and BSC. The AHP method was used to design the index weights, and the fuzzy evaluation was implemented to obtain the overall evaluation value and values of concrete indexes at all levels. In the end, the applicability and operability of this evaluation model were verified through the empirical survey of one case in Chengdu-Chongqing economic circle. Through expert judgment and data research, both subjectivity and objectivity data were collected, and then the data modeling was integrated into practical operation, this research tries to make a breakthrough in the establishment of performance evaluation index system and evaluation model of agritourism CKMCs, the final purpose was to enrich the research of customer knowledge management of tourism enterprises.

### Research methods

**Fig 1:**
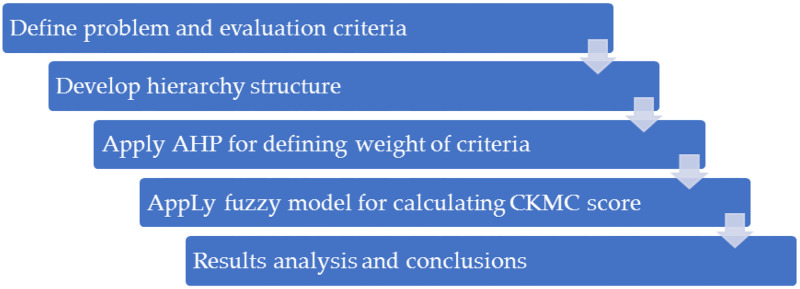
Research procedure.

This study mainly adopts fuzzy-AHP method for conducting performance evaluation of CKMC. Thus, the following part briefly introduce AHP and fuzzy set analysis in sequence. And the research process of this study is shown in Fig 1.

### AHP analysis

The analytical hierarchy process (AHP) decomposes a complex multi-attribute decision making problem into a system of hierarchies [41]. This system of hierarchies uses a pairwise comparison technique aimed at eliciting numerical evaluations of qualitative phenomena from experts and decision makers. And the following presented the procedure of AHP analysis.

#### Problem decomposition and establishment of hierarchical framework

The first step is to define the problem and construct a hierarchical framework based on index system. Based on the index system of CKM competence in the literature review, the CKMC evaluation model is then constructed. The model is divided into three layers: target layer A, criterion layer B, and index layer. The level I, II and III indexes include:

$${\text{X}}_{1}=\left({\text{X}}_{11},{\text{X}}_{12},{\text{X}}_{13},{\text{X}}_{14}\right)$$

$${\text{X}}_{2}=\left({\text{X}}_{21},{\text{X}}_{22},{\text{X}}_{23},{\text{X}}_{24}\right)$$

$${\text{X}}_{3}=\left({\text{X}}_{31},{\text{X}}_{32},{\text{X}}_{33},{\text{X}}_{34}\right)$$

$${\text{X}}_{4}=\left({\text{X}}_{41},{\text{X}}_{42},{\text{X}}_{43},{\text{X}}_{44}\right)$$

#### Construction of judgment matrix

1–9 scale method (Table 2) is used in the model, and the judgment matrix is filled by the evaluation expert group:

**Table 2:** Scale table.

Scale	Meaning
1	In the two-factor comparison, X_i_ is as important as X_j_
3	In the two-factor comparison, X_i_ is slightly more important than X_j_
5	In the two-factor comparison, X_i_ is obviously more important than X_j_
7	In the two-factor comparison, X_i_ is especially more important than X_j_
9	In the two-factor comparison, X_i_ is extremely more important than X_j_
2,4,6,8	Median of the above two adjacent judgments

The paired comparison results of four aspects—business process index, customer communication index, system support index, and market performance index—given by each expert are geometrically averaged to construct the judgment matrix A.


$$\text{A}=\left(\begin{array}{cccc} b11 & b12 & \ldots & b14\\ b21 & b22 & \ldots & b24\\ & & & \ldots \\ b41 & b42 & \ldots & b44 \end{array}\right)$$

Similarly, four judgment matrixes for the corresponding indexes at the third layer are established by taking C_i_ (i = 1,2 …,4) as the criterion.

#### Calculation of eigenvalue and eigenvector of each matrix

The eigenvectors are solved through the square root method through the following steps: the product *M*_*i*_: ${\text{M}}_{\text{i}}=\prod_{j=1}^{s}{\text{b}}_{\text{ij}}$ of factor b_ij_ in each row of B is firstly calculated, the n-order square root $\beta_{\text{i}}:\beta_{\text{i}}=\sqrt[{\text{n}}]{{\text{M}}_{\text{i}}}$ of M_i_ is then calculated, in the end, the vector β = (β_1_, β_2_,…β_n_) is normalized, namely *W*_*i*_ *= $\beta_{\text{i}}/\sum_{\text{k}=1}^{\text{s}}\beta_{\text{k}}$* is set, and then vector W = (W_1_, W_2_,…W_n_) is the solved eigenvector, and then the maximum latent root is λ_max_ = ∑W_j_ • b_ij_/W_i_.

#### Index weight and the consistency check

The single hierarchical ranking means solving the latent root of the judgment matrix A, and the eigen-vector W corresponding to the maximum latent root, and after being normalized, the two will serve as the ranking weights of the indexes regarding the relative importance to one factor in the previous layer [42]. Total hierarchical ranking refers to calculating the ranking weights of all indexes at the same level regarding the relative importance to the overall goal. In other words, it is the result by multiplying the weight of an index (third layer) in one aspect (second layer) by the weight of this aspect to the overall goal (first layer) [41, 42].

When the judgment matrix completely satisfies the following three conditions: b_ii_ = 1, b_ij_ = 1/b_ji_, and b_ij_ = b_ik_/b_jk_, then the judgment matrix is considered as complete consistency, its maximum latent root is only λ_max_ and other latent roots are all 0. In general, b_ii_ = 1 and b_ij_ = 1/b_ji_ can easily hold true, but b_ij_ = b_ik_/b_jk_ does not, requiring the following indexes during the consistency check of judgment matrix:

$$\text{C}\cdot \text{I}=\left(\lambda_{\text{max}}-\text{n}\right)/(\text{n}-1)$$ (1)

C⋅R=C⋅I/R⋅I (2)

Where R•I means Random index, which can be attained from following Table 3:

**Table 3:** R•I value.

N	1	2	3	4	5	6	7	8	9
R•I	0	0	0.58	0.89	1.12	1.24	1.36	1.41	1.45

The test criteria are as follows:

When C•R<0.1, it is considered that the judgment matrix satisfies the consistency requirement, so the ranking is effective.
When C•R≧0.1, the judgment matrix does not satisfy the consistency requirement and needs adjustment until C•R<0.1, and then a new weight table can be obtained.

### Fuzzy analysis

Fuzzy Analysis With strong fuzziness, the CKMC index of tourism enterprises can hardly be quantitatively described. As a comprehensive evaluation method based on fuzzy mathematics, the fuzzy comprehensive evaluation method was proposed by the American automatic control expert Zadel in 1965 for the first time [43]. It has great superiority in processing qualitative, uncertain, and incomplete information, so it was used in this research to evaluate the tourist satisfaction. The corresponding factors and the subfactor set as well as the corresponding weights are respectively deter-mined. The index grades should be con-firmed by combining the practical evaluation of CKMC, based on which the evaluation matrix and fuzzy evaluation model are constructed, and the fuzzy evaluation scores are calculated and the results are evaluated [43, 44]. The concrete steps are as follows:

Step 1: Determine the main factor set U and the weight, e.g. *U* = (*U*_1_, *U*_2_, *U*_3_, *U*_4_).
Step 2: Determine the factor evaluation index set and the weight, e.g. *U*_1_ = (*U*_11_, *U*_12_, *U*_13_, *U*_14_).
Step 3: Determine the evaluation grade of each index.
Step 4: Determine the evaluation matrix.

$$R=\left(\begin{array}{cccc} r_{11} & r_{12} & .. & r_{1n}\\ r_{21} & r_{22} & .. & r_{2n}\\ .. & & .. & \\ r_{n1} & r_{n2} & .. & r_{nn} \end{array}\right)$$

$${\text{r}}_{\text{ij}}=\;\left(\text{i}=1,2,\;\ldots \text{n};\text{j}=1,2,\ldots \text{m}\right)$$

r_ij_ = (i = 1,2,…n; j = 1,2,…m) denotes the membership for the index U_i_ to the evaluated as W_i_

Step 5: Construct the fuzzy evaluation model. Conduct the fuzzy matrix operation for the evaluation matrix R of indexes at the sub-factor index layer firstly, thus obtaining the member-ship vector of index relative to the evaluation set. Secondly, implement the fuzzy matrix calculation of R, followed by the normalization to obtain the membership vector B of index relative to the evaluation set.
Step 6: Calculate the fuzzy evaluation score. Calculate the evaluation score given by each evaluation subject to each index.

B=W×R (3)

The comprehensive score of each index is then calculated as below:

E=B×H (4)

Step 7: Evaluate the results. The total evaluation score is obtained by multiplying the weight value of each index by the comprehensive score of each index.

### Combination of AHP with fuzzy analysis

Modern tourism enterprises have paid increasingly high attention to the customer knowledge management, which is the integration of customer relation-ship management and knowledge management [2, 3]. However, the evaluation of enterprise CKMC has not been extended in depth or breadth, and its combination with the practical application in enterprises is not close enough. In order to realize the objective and accurate evaluation of enterprise CKMC, the AHP and fuzzy evaluation method were used to construct the comprehensive evaluation model of CKMC performance, and its applicability and reliability were verified by case study. The research conclusions were drawn as follows:

1. The AHP and fuzzy evaluation method-based CKMC evaluation model can mitigate the influences of subjective factors and uncertainty on the evaluation result, and improve the evaluation reliability. When the AHP method is used to deter-mine the index wrights, the human subjective judgment is expressed and processed in quantitative form, trying to reduce the draw-backs brought by individual subjective speculation; the fuzzy comprehensive evaluation can relieve the adverse effects of uncertainty and fuzziness on the evaluation result to some ex-tent [45]. The advantages of the two methods were integrated in this research to improve the authenticity and accuracy of the evaluation result.
2. The AHP and fuzzy evaluation method-based CKMC evaluation model can identify the key factors influencing the enterprise CKM and their potential mechanisms very well. Among the lev-el I indexes, enterprise CKM process and system support are the key factors, and customer communication and sales performance are representational influence factors; among the Lev-el II indexes, customer knowledge sharing and application, timeliness of customer information exchange, and importance attached by the senior leadership contribute considerably to the CKMC scores.
3. The AHP and fuzzy evaluation method-based CKMC evaluation model is of high practical application value. The tourism enterprises can seek for the paths to improve their CKMC according to this model. Based on the evaluation index system, the enterprises can review their CKM operation status from overall perspective and multiple dimensions, making up their deficiencies and shortcomings, improve their customer knowledge application capabilities, and strengthen their innovation capabilities.

All in all, the evaluation method, which combines the AHP and fuzzy comprehensive evaluation method, integrates the advantages of quantitative and qualitative analyses. The AHP method is used to decompose the human thinking process into layers, the weights of evaluation indexes are solved, and then the fuzzy evaluation method is used to trans-form the fuzzy evaluation results in the questionnaires into quantitative degree of membership, thus improving the authenticity and objectivity of the evaluation result to the greatest extent [45, 46]. However, the interaction between evaluation indexes and quantitative processing of subjective decisions remain to be further investigated and deepened.

## Empirical study

### Sample description and survey implementation

New Hope Modern Agritourism Development Co. LTD (NHMA) is a full member of Sichuan New Hope Group and the president unit of Chongqing Chamber of Tourism. As a leading tourism company with considerable influence in Chengdu-Chongqing economic circle, this enterprise owns a marketing network system stretching all over Chengdu-Chongqing economic circle. With 67 sales networks and over 1,000 employees in Chongqing [47], it possesses rather abundant tourism products, including tourist attractions development, tourism facilities sales, leisure and agritourism, Land consolidation, Health tourism, Elderly care services, etc. [43].

An empirical survey was conducted from Feb 20^th^ to 27^th^ of 2022 within various departments of New Hope Modern Agritourism Development Co. LTD, and a questionnaire method was adopted for collecting experts’ and employees’ perceived value of CKMC. Before the survey, the participants were informed that this survey was used to collect employees’ individual feeling and subjectively evaluation about organizational CKMC, the data were collected and analyzed anonymously for guaranteeing valid and privacy, and furthermore only those respondents provided their verbal consent were recruited to this study. All the respondents participated in this survey voluntarily.

Finally, A total of 350 questionnaires were given out, 345 ones were recovered, and through the validity check, there were 320 valid questionnaires, and the effective rate was 91.4%; in addition, the expert symposiums (6 experts) and interview (50 participants) were held for four times in this survey.

The respondents included the staffs in the outlets in 19 districts and counties like Yuzhong District, Jiangbei District, and Shapingba District, and they were basically young and middle-aged, accounting for 52.50% of total number of respondents. The employees presented little gender difference, female employees accounted for 54.37%, and female employees for 45.63%. The respondents were mainly shop assistants and shop managers who accounted for 53.12% and 19.37%, respectively. The experts invited to the symposium mainly came from colleges and universities, governmental sectors, and third-party customer resource management training institutions.

### AHP analysis

After data collection, the data were firstly sorted and analyzed by AHP method.

#### Establishment of judgment matrix and determination of the index weight

**Fig 2:**
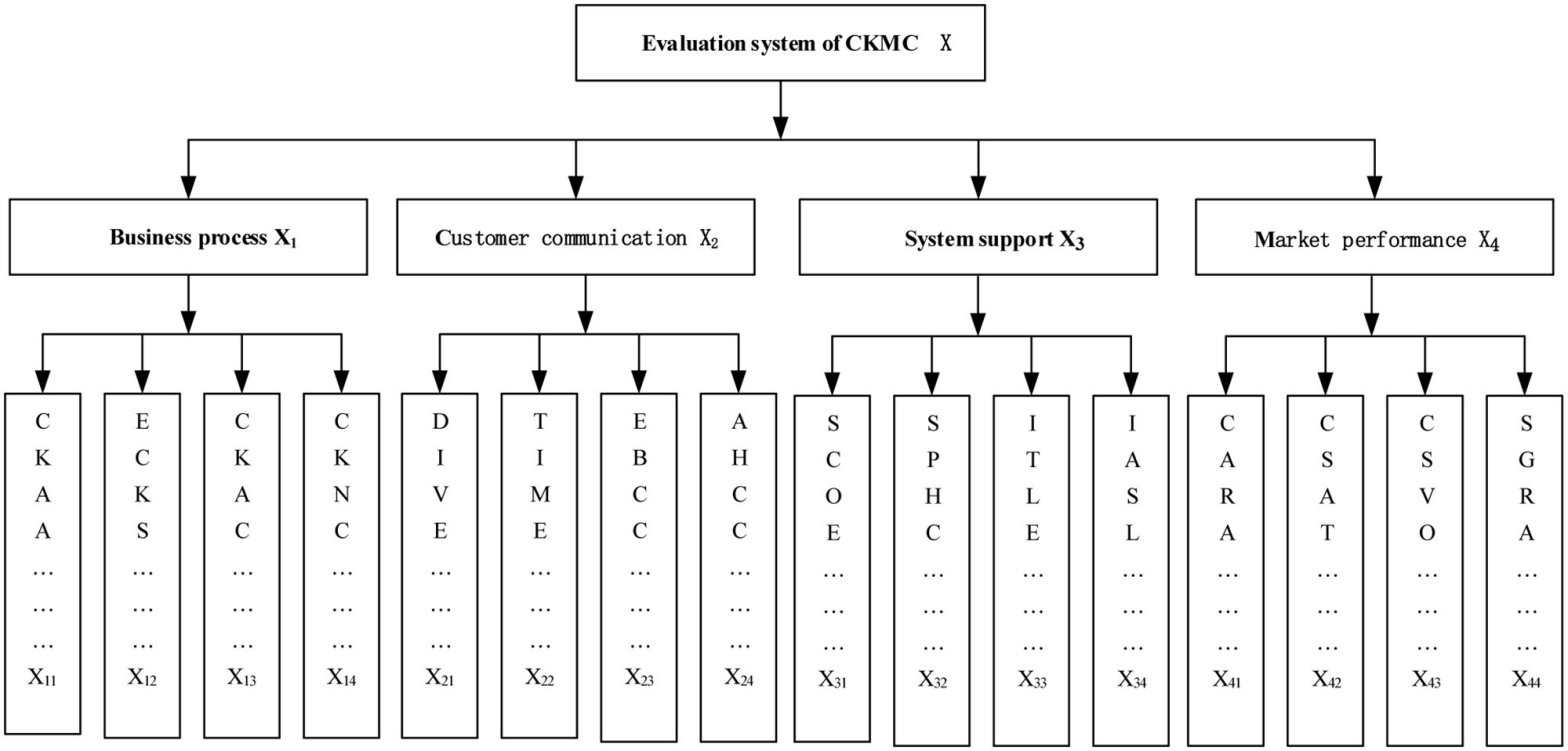
Evaluation system of CKMC.

The judgment matrix of index weights was con-structed according to the index system in Fig 2. In the expert interview (Delphi method), the questionnaires and actual suggestions regarding the judgment of index weights were collected from 6 experts. The data processing of the judgment matrix was con-ducted via the AHP process. The calculation process was explained by taking the judgment given by an expert for example. The judgment matrix of level I indexes for NHMA CKMC evaluation was obtained according to the experience-based judgment of this expert as seen in Table 4.

**Table 4:** Judgment matrix of level I indexes of CKMC evaluation.

Title 1	Business process	Customer communication	System support	Market performance
Business process	1	3	2	5
Customer communication	1/3	1	1/2	2
System support	1/2	2	1	3
Market performance	1/5	1/2	1/3	1

#### Calculation of product and n-order square root of judgment matrix

The product of factors in each row of the judgment matrix is firstly calculated, and then:

$${\text{M}}_{1}=\;30,{\text{M}}_{2}=0.333,{\text{M}}_{3}=3,{\text{M}}_{4}=0.033$$

Secondly, the n-order square root is calculated. The n-order square root of Fi is calculated. $\beta_{\text{i}}=\sqrt[{\text{n}}]{{\text{M}}_{\text{i}}}$ and n = 4 is set, then:

$$\beta_{1}=2.340,\beta_{2}=0.760$$

$$\beta_{3}=1.316,\beta_{4}=0.427$$

#### Index weight and consistency check

First, the vector (β_1_, β_2_, β_3_, β_4_)^T^ = (2.340, 0.760, 1.316, 0.427)^T^ is normalized, and the weights of Level I indexes for the CKMC evaluation can be obtained as: W1 = 0.483, W2 = 0.157, W3 = 0.272 and W4 = 0.088.

Second, the consistency check is implemented. The maximum eigenvalue of the judgment matrix is firstly calculated, followed by the calculation of consistency indexes C•I and C•R, and thus λmax = 4.014; C•I = 0.005. According to Tab. 2, R•I = 0.89 under n = 4, C•R = C•I/R•I = 0.005/0.89 = = 0.006< 0.1, indicating that this judgment matrix is of satisfying consistency.

The vector W = (0.483, 0.157, 0.272, 0.088)^T^ can serve as the weight vector. The other level I indexes and level II indexes are processed in a way similar with the above. The mean value if calculated by selecting the weight values given by the 6 experts, and the consistency check is passed. The weights of level I and II indexes for the CKMC evaluation of this enterprise are listed in Table 5.

**Table 5:** Weights of index factors.

Target layer	Level I index	Weight	Level II index	Weight
Evaluation system of CKMC	Business process	0.465	CKAA	0.193
			ECKS	0.366
			CKAC	0.319
			CKNC	0.122
	Customer communication	0.152	DIVE	0.117
			TIME	0.372
			EBCC	0.213
			AHCC	0.298
	System support	0.289	SCOE	0.055
			SPHC	0.258
			ITLE	0.311
			IASL	0.376
	Market performance	0.094	CARA	0.406
			CSAT	0.298
			CSVO	0.105
			SGRA	0.191

### Fuzzy analysis

#### Weight analysis of attributes

Firstly, the evaluation set V = (V_1_, V_2_, V_3_, V_4_, V_5_) = (very satisfied, satisfied, generally satisfied, dissatisfied, very dissatisfied) = (5,4,3,2,1) is established; the evaluation index set U for CKMC of tourism enterprise includes four level I indexes: business process, customer communication, auxiliary support, and market performance, so U = (i = 1,2,3,4), where each U_i_ consists of four level II indexes U_ij_, namely U = U_ij_.

According to Tab. 5, the weights of level I and II indexes are presented as follows:

F = (0.465, 0.152, 0.289, 0.094)
B = (0.193, 0.366, 0.319, 0.122)
C = (0.117, 0.372, 0.213, 0.298)
D = (0.055, 0.258, 0.311, 0.376)
E = (0.406, 0.298, 0.105, 0.191).

#### Fuzzy evaluation analysis of CKMC

Secondly, according to the CKMC evaluation index system (Fig 2), the ratio of number of respondents with each index belonging to the evaluation set V to the total number of respondents is obtained, that is, Ri (i = 1,2,3). The evaluation matrixes of level II indexes are calculated using the fuzzy comprehensive evaluation model as follows:

$${\text{R}}_{1}=\left(\begin{array}{ccccc} 0.219 & 0.368 & 0.201 & 0.195 & 0.017\\ 0.101 & 0.251 & 0.198 & 0.375 & 0.075\\ 0.281 & 0.369 & 0.263 & 0.075 & 0.013\\ 0.163 & 0.219 & 0.412 & 0.186 & 0.020 \end{array}\right)$$

$$R_{2}=\left(\begin{array}{ccccc} 0.335 & 0.389 & 0.182 & 0.079 & 0.015\\ 0.413 & 0.315 & 0.161 & 0.111 & 0.000\\ 0.189 & 0.298 & 0.365 & 0.121 & 0.027\\ 0.288 & 0.319 & 0.338 & 0.042 & 0.013 \end{array}\right)$$

$$R_{3}=\left(\begin{array}{ccccc} 0.293 & 0.318 & 0.279 & 0.110 & 0.000\\ 0.421 & 0.310 & 0.269 & 0.000 & 0.000\\ 0.124 & 0.169 & 0.457 & 0.216 & 0.034\\ 0.092 & 0.196 & 0.387 & 0.141 & 0.184 \end{array}\right)$$

$$R_{4}=\left(\begin{array}{ccccc} 0.201 & 0.315 & 0.296 & 0.126 & 0.062\\ 0.192 & 0.295 & 0.287 & 0.134 & 0.092\\ 0.189 & 0.301 & 0.298 & 0.124 & 0.088\\ 0.216 & 0.245 & 0.301 & 0.129 & 0.109 \end{array}\right)$$

According to Eq (3) and the index weights, the fuzzy comprehensive evaluation sets for the level II indexes are calculated as follows:

$${\text{B}}_{1}={\text{W}}_{1}\times {\text{R}}_{1}=\;\left(0.189,\;0.307,\;0.245,0.222,0.037\right)$$

$${\text{B}}_{2}={\text{W}}_{2}\times {\text{R}}_{2}=\;\left(0.319,0.321,0.260,0.089,\;0.011\right)$$

$${\text{B}}_{3}={\text{W}}_{3}\times {\text{R}}_{3}=\;\left(0.198,\;0.224,0.372,0.126,0.080\right)$$

$${\text{B}}_{4}={\text{W}}_{4}\times {\text{R}}_{4}=\;\left(0.200,0.294,0.294,0.129,0.083\right).$$

The deblurring operation is done for the evaluation set at each criterion layer according to Eq (4), and then the evaluation values of business process, customer communication, system support, and market performance are obtained:

$${\text{E}}_{1}=5{\text{b}}_{11}+4{\text{b}}_{12}+3{\text{b}}_{13}+2{\text{b}}_{14}+{\text{b}}_{15}=3.389$$

$${\text{E}}_{2}=5{\text{b}}_{21}+4{\text{b}}_{22}+3{\text{b}}_{23}+2{\text{b}}_{24}+{\text{b}}_{25}=3.848$$

$${\text{E}}_{3}=5{\text{b}}_{31}+4{\text{b}}_{32}+3{\text{b}}_{33}+2{\text{b}}_{34}+{\text{b}}_{35}=3.334$$

$${\text{E}}_{4}=5{\text{b}}_{41}+4{\text{b}}_{42}+3{\text{b}}_{43}+2{\text{b}}_{44}+{\text{b}}_{45}=3.399$$

The final evaluation set for the CKM level is obtained through the fuzzy comprehensive evaluation method:

$$\text{A}=\text{W}\times \text{B}=\;\left(0.212,0.284,0.289,0.165,0.050\right)$$

The deblurring operation is implemented for the final evaluation set, and the comprehensive evaluation of tourist satisfaction is obtained:

E=5×0.212+4×0.284+3×0.289+2×0.165+0.050=3.443

To sum up, the scoring results of the evaluation indexes for the CKMC of NHMA are seen in Table 6.

**Table 6:** Final score of CKMC.

Target layer	Level I index	Weight	Level II index	Weight
Final score of CKMC (3.443)	Business process	3.389	CKAA	3.577
			ECKS	2.928
			CKAC	3.833
			CKNC	3.319
	Customer communication	3.848	DIVE	3.950
			TIME	4.030
			EBCC	3.501
			AHCC	3.827
	System support	3.334	SCOE	3.794
			SPHC	4.152
			ITLE	3.133
			IASL	2.871
	Market performance	3.399	CARA	3.467
			CSAT	3.361
			CSVO	3.379
			SGRA	3.330

## Results analysis and discussions

Based on above AHP and fuzzy analysis, it can be seen that (1) The overall CKMC score of this group is 3.443.According to maximum membership principle, this score falls in this interval between “generally satisfied” and “satisfied”, indicating that the workers at frontline employees and grassroots operating departments are basically satisfied with the CKMC of Chongqing NHMA ltd. (2) as to the level-I index, the score of these four dimensions from high to low were: Customer communication dimensions (3.848), Market performance dimension (3.399), Business process performance dimensions (3.389), and System support dimensions (3.334), all these scores were higher than 3, indicating that most of them are basically satisfied with CKMC practice in this group, among them customer communication dimensions was much higher than others, this reflected that CKM indeed enhance the communication toward customers.

As to the level-II index, (1) in the customer communication dimension, the score of such factors including timeliness of customer communication, diversity of communication channels, and ability to handle customer com-plaints are 4.030, 3.950, and 3.827, respectively, higher than the average level. whereas the score of communication efficient was just 3.501, lower than the former ones. As to the reasons, it may be ascribed to the redundant organizational structure, lack of authorization among front-line employees, and unsmooth internal communication mechanism. In subsequent, (2)The evaluation value of system support is 3.334, which is lower than the overall evaluation index of this group, where the mini-mum degree of importance attached by senior leadership is 2.871, manifesting that the senior leadership has not fully recognized the strategic significance of CKM or profoundly understand the operating mechanism of CKM; the evaluation value of information technology level is 3.133, indicating that the information technology has not systematically exerted its potential effect on facilitating the enterprise CKM; the evaluation value of organizational environment is 3.794, which is mainly ascribed to overstaffing of state-owned enterprise, overlapping departments, and difficult effective knowledge flow (3) The evaluation value of business process is 3.389, where those of customer knowledge acquisition capability, customer knowledge application capability, and customer knowledge innovation capability are 3.577, 3.833, and 3.319, respectively, which basically satisfy the related requirements, but the score of customer knowledge sharing mechanism is only 2.928, meaning that the customer knowledge is not shared in the effective communication of the enterprise, and this is associated with low scores of degree of importance attached by the senior leadership (2.871), and supporting capacity of organizational environment (3.794), and these, together, contribute to the low score of CKMC in New Hope Group. (4) The score of market performance is 3.399, which is just around the average level of whole company. within this dimension, the score of customer acquisition rate (CARA), customer satisfaction, cross-sales volume, and sales growth rate are 3.467, 3.361, 3.379 and 3.330 respectively, this indicated that CKM directly increase the customer acquisition rate while the influence on market performance is relatively low. as to the reason, low level of infrastructure construction together with less attention paid from the leaders, leads to the low implementation level of CKMC, and not to mention its connected effect on market development and financial revenue.

In addition, among all the 16 sub-factors, only 2 factors reach above 4.0, and they are timeliness of communication (4.030) and sustaining power of human resource (4.152). The index factors with the lowest scores were degree of importance attached by the senior leadership (2.871), efficiency of customer knowledge sharing mechanism (2.928), and information technology level (3.133), respectively, indicating that the enterprise should focus on the transition of leadership concept and organizational structure design.

## Conclusions and implications

### Conclusions

CKM has been in operations for many years, literature suffers from a lack of understanding of customer knowledge management competence evaluation, especially in traditional agritourism industry. Similar in China, although agritourism has been a hot issue in the past two decades, neither the management or stockholders of agritourism business can fully understand the factors and attributes influencing CKM long-term business performance [48]. Previous studies have confirmed the importance of key attributes in numerous management fields. thus, it is necessary to identify the key performance indicators of CKM and define their degree of importance.

The purpose of this paper was to establish the performance evaluation index for customer knowledge management within agritourism industry by using balanced scorecard methods and fuzzy AHP. Through literature review and expert opinions, four main index dimensions for CKMC evaluation were constructed, which included Business process performance dimensions, Customer communication dimensions, System support dimensions, and Market performance dimensions. Empirical results show that the relative importance of these four dimensions from high to low were: Business process performance dimensions (0.465), System support dimensions (0.289), Customer communication dimensions (0.152) and Market performance dimension (0.094). Furthermore 16 sub-factors were also established from these four aspects. Business process performance measurement indicators were ranked in order of importance as ECKS, CKAC, CKAA and CKNC. The System support measurement indicators were IASL, ITLE, SPHC, and SCOE, in order of importance. The Customer communication measurement indicators are ranked, in order of importance, as TIME, AHCC, EBCC and DIVE. The performance measurement indicators for market performance are CARA, CSAT, SGRA, and CSVO, in order of importance. Taking New Hope Modern Agritourism Development (Group) Co., Ltd as the example, the empirical study shows that the overall score of CKMC within NHMA is 3.443, which means generally satisfied. it also reflected that respondents were generally satisfied with NHMA CKMC. and as to the level I index, the score of these four dimensions from high to low were: Customer communication dimensions (3.848), Market performance dimension (3.399), Business process performance dimensions (3.389), and System support dimensions (3.334), this reflected that respondents were much more satisfied with the customer communication and market performance brought by customer knowledge management. As to the 16 sub-factors, the top 3 high ones were SPHC, TIME and DIVE, the latter two ones were classified into customer communication, this also proves that CKM brings much more benefits to business customer communication in general.

This research has the following contributions to CKM theory and business practice. firstly, CKM has been widely applied in business management for almost 20 years, while it most applied in the high-technology manufacturing industry such as information and communication company, the application and evaluation of CKM in traditional agritourism industry is relatively few. Nowadays under the context of information age, with the assistance of digital economy and smart tourism, the application and evaluation of CKM becomes necessary. Therefore, as far as the industrial application is concerned, it is very important to construct evaluation index and conduct objective evaluation of traditional CKM application and digital transformation. secondly although there are quite a number of researches discussing the application and implementation of CKM, relatively few publications dealing with performance evaluation of CKMC within traditional industry [8]. Thirdly, the balanced scorecard has been adopted for measuring performance indicators in strategy management, but few researches have applied it in knowledge management field. This study uses a balanced scorecard to measure CKMC of agritourism enterprise, it is innovation in the application of traditional method. Fourthly, by using fuzzy AHP method to verify the validity of experts and empirical study is also an objective and rigor verification method that has been affirmed academically.

### Recommendation and implications

Based on the analysis above, results showed that while implementing customer knowledge management, customer communication, and system support should be put on first priority as they ranked top 2 on the dimension list. Thus, both academic and practitioners should strengthen mutual communication with customers and strengthen the informational infrastructure construction for information exchange. while on the other hand, empirical study demonstrated that lack of attention from leadership, inefficiency of customer knowledge sharing together with low-level of information technology have indeed hindered the sharing and communication of customer knowledge. Thus, industry practitioners should pay much attention leadership development and organizational redesign. leaders especially top management should pay much attention on the implementation of CKM, and flexible organization should also be adopted for enhancing information exchange and information flow [49].

As to the academic filed, First, follow-up studies need to further broaden the indicator design, improve the compatibility and tolerance of evaluation indicators to provide reference and reference for the construction of CKMC performance evaluation index. Second, on the selection of performance appraisal methods, more appropriate performance appraisal systems can be selected. In terms of research methods, other data analysis methods can be used to demonstrate and analyze the evaluation indicators in order to increase the comprehensiveness of the argument and reliability.

## Supporting information

S1 Data CKMC data. (XLSX)
